# Path loss modeling based on neural networks and ensemble method for future wireless networks

**DOI:** 10.1016/j.heliyon.2023.e19685

**Published:** 2023-09-06

**Authors:** Mohamed K. Elmezughi, Omran Salih, Thomas J. Afullo, Kevin J. Duffy

**Affiliations:** a The Discipline of Electrical, Electronic and Computer Engineering, University of KwaZulu-Natal, Durban, 4041, South Africa; b Institute of Systems Science, Durban University of Technology, Durban, 4000, South Africa

**Keywords:** Wireless communications, Channel modeling, Path loss, Neural network, ANN, RNN-LSTM, CNN, Ensemble method, 5G, 6G

## Abstract

In light of the technological advancements that require faster data speeds, there has been an increasing demand for higher frequency bands. Consequently, numerous path loss prediction models have been developed for 5G and beyond communication networks, particularly in the millimeter-wave and subterahertz frequency ranges. Despite these efforts, there is a pressing need for more sophisticated models that offer greater flexibility and accuracy, particularly in challenging environments. These advanced models will help in deploying wireless networks with the guarantee of covering communication environments with optimum quality of service. This paper presents path loss prediction models based on machine learning algorithms, namely artificial neural network (ANN), artificial recurrent neural network (RNN) based on long short-term memory (LSTM), shortly known as RNN-LSTM, and convolutional neural network (CNN). Moreover, an ensemble-method-based neural network path loss model is proposed in this paper. Finally, an extensive performance analysis of the four models is provided regarding prediction accuracy, stability, the contribution of input features, and the time needed to run the model. The data used for training and testing in this study were obtained from measurement campaigns conducted in an indoor corridor setting, covering both line-of-sight and non-line-of-sight communication scenarios. The main result of this study demonstrates that the ensemble-method-based model outperforms the other models (ANN, RNN-LSTM, and CNN) in terms of efficiency and high prediction accuracy, and could be trusted as a promising model for path loss in complex environments at high-frequency bands.

## 1 Introduction

Rapid developments in implementing Internet of Things (IoT) networks have occurred in recent years, owing primarily to improved communication and sensing capabilities paired with lower IoT device pricing [1]. As a result, it is expected that, according to the Cisco forecast, the number of IoT-connected devices will be more than 500 billion by 2030. Moreover, a threefold increase in annual global data traffic is expected in the next five years [2].

The power of a wireless signal rapidly decays as the distance between the transmitting and receiving antennas increases (even in free space). This fundamental concept in wireless communications is called path loss, a vital factor for coverage prediction and radio frequency (RF) planning of any wireless system. Path loss behavior mainly depends on distance, frequency, and the propagation environment where the transmitter and receiver are placed [3], [4].

Several crucial parameters in wireless communications, such as throughput, signal-to-noise ratio, probability of error, and many other metrics, are determined by propagation channel modeling. Hence, accurate channel modeling can lead to the optimum planning of wireless networks [3].

The focus of this paper is on predicting the path loss due to it being the main propagation channel factor for designing wireless systems since the overall network performance is highly affected by path loss [5], [6].

Many researchers in the field of propagation path loss modeling have adopted models where the path loss decays with the distance raised to the power *n*, where *n* is well-known as the path loss exponent (PLE). However, the PLE values vary significantly depending on the environment where the wireless system is placed and the communication scenario. The log-distance model is a widely used empirical model for predicting path loss [7], [8], [9], [10], [11], [12], [13], [14]. This model assumes a linear relationship between path loss and the distance between the transmitter and receiver on a logarithmic scale. The proportional parameter value(s) are obtained through linear regression analysis of measurement data collected from campaigns or simulation tools. However, despite being straightforward and easy to use, this model may only accurately predict path loss in some radio propagation scenarios. As a result, advanced modeling techniques are necessary for predicting path loss more accurately and flexibly in complex and diverse environments. To achieve this, some researchers have proposed improvements to the log-distance model, such as incorporating the impact of shadowing and other factors like antenna height, operating frequency, clutter, terrain, communication scenario category (i.e., line-of-sight (LOS) or non-line-of-sight (NLOS), etc.), and the percentage of built-up areas covered by buildings [8], [9], [10], [11], [12], [13], [14], [15].

Because of ground reflections, scattering, blocking/shadowing, and other physical factors, propagation in terrestrial situations is substantially difficult to quantify [16]. Accordingly, owing to the complexity of some actual propagation environments, the traditional empirical models will not be precise for complex and vital environments. Moreover, this problem will continue to grow since future wireless communication systems will exploit the millimeter- wave (mmWave) and sub-terahertz (sub-THz) frequencies, and higher frequency bands to meet future data needs (with the help of advanced technologies).

One of the promising solutions to achieve the most accurate propagation models is to use or develop machine learning (ML) algorithms. ML algorithms have massively attracted both the academia and industrial worlds due to their efficiency and high prediction accuracy over the traditional methods. For example, but not limited to, ML methods were successfully exploited in several wireless communication systems, such as wireless body area networks (WBANs) [17], vehicular communication networks [5], smart metering systems [18], modulation detection [19], channel estimation [20], [21], channel encoding and decoding [22], [23], path loss prediction [15], [24], [25], [26], [27], [28], [29], [30], [31], [32], [33], [34], [35], [36], and many others.

A path loss prediction model for vehicular-to-vehicular communications using a random forest has been evaluated in terms of accuracy and generalization capability [5]. The random forest method has been successfully used in various aspects due to its simplicity and relatively high prediction accuracy. Path loss prediction using artificial neural networks for cellular networks with wireless channels between base stations and users is discussed in [31], [34]. In addition, the authors in [37] propose a multi-kernel-based online path loss prediction model incorporating trajectory information and user location for the downlink. Air-to-Ground path loss modeling in urban environments has been presented in [38] for unmanned aerial vehicles (UAVs) applications. The UAVs recently are of vital interest in different domains due to their ease of feasibility and mobility. The ML models proposed in [38] are based on artificial neural networks (ANN), regression trees (RT), and K-nearest-neighbors (KNN). A similar performance was observed by the three models in three selected frequency bands of 433, 900, and 5800 MHz, with a slight outperformance of the KNN model since lower values of both the root-mean-square error (RMSE) and mean absolute error (MAE) were achieved compared to the other two models.

A feed-forward deep neural network (DNN) model is presented and compared with the well-known traditional alpha-beta-gamma (ABG) model over a wide range of frequencies between 0.8 and 70 GHz for suburban and urban environments in [24]. The DNN model, as expected, provided better performance than the ABG model since better performance metrics were achieved, such as the mean-square error (MSE) was improved by 6 dB. The study also exploited the XGBoost algorithm for the evaluation of the contribution of the input features to the prediction accuracy of the proposed model [24]. More studies in the literature that are focused on path loss prediction based on ML algorithms can be found in [15], [25], [29], [39], [40], [41], [42], and references therein.

There is a severe lack of literature on predicting path loss using more advanced ML algorithms. This is because the existing research relied mainly on well-known ML methods and adjusted the models to fit measurement data with minimum errors. In comparing these ML-based and empirical models, ML methods have proved their superiority in terms of accuracy since they can provide complex equations to describe the path loss compared to the empirical models that mainly utilize a few parameters.

The main objective of the current paper is to provide the most accurate and stable machine-learning-based path loss prediction model. To this end, we propose and evaluate the performance of a neural network model based on the ensemble method. The model is based on three neural network models: artificial neural networks (ANN), artificial recurrent neural networks (RNN) based on long short-term memory (LSTM), shortly known as RNN-LSTM, and convolutional neural networks (CNN). The ensemble-method-based model is compared with the three separate models using various well-known evaluation metrics to prove its superiority among these models. The reason for choosing these neural network models as the main parts of the proposed model is the fact that they provide the most accurate performance compared to several machine-learning-based models, including multiple linear regression (MLR), polynomial regression (PR), support vector regression (SVR), decision trees (DT), random forests (RF), and K-nearest neighbors (KNN), as found in a comparative analysis published in [43].

Moreover, combining the strengths of different machine learning models can lead to better performance on a given task. This is the idea behind ensemble learning, where multiple models are trained, and their predictions are combined in some way to make a final prediction. The proposed ensemble model uses ANN, RNN-LSTM, and CNN models based on their strengths and their ability to complement each other's weaknesses. For example, feedforward neural networks are good at modeling structured and tabular data but need to be better suited for handling sequential data like time series or natural language. RNN-LSTM, on the other hand, are specifically designed to process sequential data and can learn long-term dependencies, but they can be challenging to train and may suffer from vanishing gradients. CNNs, on the other hand, are particularly effective at processing data with a grid-like structure, such as images, and can learn local patterns and features. However, they could be more effective at modeling sequential data or long-term dependencies. Combining these models of neural networks makes it possible to take advantage of their strengths and overcome some of their weaknesses mentioned above. By combining ANNs, RNN-LSTMs, and CNN's, ANNs can learn complex relationships between the inputs and outputs, RNN-LSTMs can model dependencies between time steps, and CNN's can detect patterns in spatial data. Together, these models can provide a more robust and accurate prediction than any single model alone [44], [45], [46].

To the best of our knowledge, based on a literature review, this is the first effort that uses ensemble-method-based neural networks for predicting the path loss. All the existing studies exploit only separate algorithms for this objective. Moreover, the model run time is used to evaluate the proposed model's performance and compare it with other standard ML algorithms. Also, the relative contribution of the models' input features to prediction accuracy can be used to carefully consider when collecting the raw data via measurement campaigns, drive tests, or simulation tools, as well as in building any ML-based model for similar environments. The data used in this research was collected in a typical indoor corridor environment at three selected frequency bands, namely 14 GHz, 18 GHz, and 22 GHz. Two communication scenarios were considered during the measurement campaigns: line-of-sight (LOS) when both antennas are aligned toward each other with no obstacle in between, and non-line-of-sight (NLOS) when the receiving antenna relies only on the propagation mechanisms to receive the transmitted signals. Details about the measurement campaigns used can be found in [4], [47].

The remainder of the current paper is structured as follows. First, the data pre-processing technique used and the adopted evaluation metrics are provided in section 2. After that, section 3 introduces in detail the proposed ensemble-method-based neural network model and its structure based on the ANN, RNN-LSTM, and CNN models. Then, the main research findings of this paper are presented and discussed in section 4. Finally, section 5 concludes the paper and gives insights into the future scope of this research.

## 2 Data preparation and evaluation metrics

### 2.1 Measurement setup and data collection method

This section provides a detailed account of the radio frequency propagation measurement campaigns carried out in a typical enclosed corridor environment located on the 5th floor of the Discipline of Electrical, Electronic, and Computer Engineering at the University of KwaZuluNatal, Howard College Campus, Durban 4001, South Africa. To ensure accuracy, the researchers carefully calibrated the measurement system and made sure that no other transmissions on the same radio frequency bands were present before beginning the campaigns. The corridor environment has dimensions of 30, 1.4, and 2.63 meters in length, width, and height, respectively, with both sides consisting of bricks and dry concrete with wooden doors. Noteworthy is the fact that indoor corridors like this can be estimated as rectangular air-filled waveguides with large sizes compared to signal wavelengths, making this measurement setup ideal for many indoor applications.

For this experiment, we used vertically polarized antennas with directional radiation patterns. The Tx antenna was mounted at two different heights, 160 centimeters and 230 centimeters above the floor level, while the Rx antenna was positioned at the height of 160 centimeters. These antenna heights are commonly adopted by researchers for indoor environments [42], [48], [49], [50]. In situations where the Tx antenna was elevated to 230 centimeters. To make sure that both antennas' boresights were aligned for all measurements in the LOS communication scenario, we down-tilted the antenna. We also considered three SHF band frequencies for this study, namely 14, 18, and 22 GHz. Pyramidal horn antennas were utilized, and at the operational frequencies, they had directional gains of between 19.5 and 22.1 dBi with half-power beamwidth values ranging from 13 to 19.2 degrees. Throughout the measurement campaigns, the Tx antenna was placed at one end of the corridor, and the Rx antenna was moved incrementally away from the Tx antenna in steps of 2 meters at a time, with the Tx-Rx separation distance ranging from 2 to 24 meters. According to the advice of most specialists in this sector, the reference Tx-Rx distance was fixed at 1 meter [51], [52], [53]. To satisfy the far-field criteria, it should be remembered that the distance from the transmitter (Tx) must be significantly longer than the wavelength of the lowest operational frequency. This criterion was previously met because the SHF signals' wavelengths are in the millimeter range.

A distinction was made between measurements taken under LOS and NLOS communication scenarios. In the LOS scenario, the antennas were directly aligned, and there were no obstructions in the direct path between them. However, in the NLOS scenario, the antenna alignment was not on boresight, and the received signal was affected by diffraction, reflection, and waveguiding mechanisms in the corridor environment. The transmit power remained constant at 10 dBm during the measurements, while the received power level ranged from −41.33 to −19.05 dBm. The angle of departure (AoD) was fixed at 0 degrees, while the angle of arrival (AoA) was varied in increments of 10 degrees from 0 to 360 degrees.

Measurement data collected from campaigns includes 865 at various frequencies, Tx-Rx distances, antenna heights, and AoA values. We cleaned and analyzed the raw data to obtain a reliable path loss value for each combination of parameters. The four key input features for the ML-based models used in this study are the Tx-Rx distance, operating frequency, AoA of the Rx antenna, and Tx antenna height, which are important factors affecting the path loss values. These features were selected to develop multi-frequency path loss prediction models that can generalize well.

The collected raw datasets underwent analysis and cleaning to obtain a reliable path loss value for each frequency, Tx-Rx separation distance, AoA, and Tx antenna height. Four features were identified as the most significant for optimal performance of the ML-based models used in this study. Firstly, the distance between the transmitting and receiving antennas, which has a significant impact on path loss values. Secondly, the operating frequency to enable multi-frequency path loss prediction models between 14 and 22 GHz. Thirdly, the AoA of the Rx antenna to provide LOS and NLOS communication characterizations. Finally, the Tx antenna height to allow for more generalization of the target models.

It is important to note that for this study, the proposed model was trained on 80% of the data, while 20% of the data was reserved for testing. All of the results presented in the tables and figures are based on the model's performance on the test data. This is a common practice in machine learning to evaluate the generalization ability of the model, that is, its ability to make accurate predictions on unseen data. Using a separate test set allows us to assess the model's performance on data it has not seen during training, which helps to ensure that the model is not overfitting to the training data. It is also essential to use a representative sample of the data for testing to ensure that the results accurately reflect the model's performance on the entire dataset.

### 2.2 Data pre-processing

The measured dataset has been pre-processed using three techniques: data cleaning, normalization, and reduction. Firstly, the data measured was cleaned due to the importance of this step in ML-based modeling. Training ML-based models with impure datasets can lead to various severe problems. In contrast, meticulously cleansed and superior datasets lead to a model that can produce exceptional outcomes. There are many data-cleaning methods used in the literature. This study has employed a technique to eliminate irrelevant data and handle missing values through interpolation that involves estimating the missing values by interpolating between the nearest two available values.

Secondly, the selected input features are normalized. The normalization of the cleaned measured data is acquired for processing the learning tasks. The adopted ML models were trained and tested using the normalized data. To achieve this, the initial step was to calculate the mean value for each feature, followed by subtracting this mean value from the corresponding feature value of the entire dataset to center the data. Subsequently, the standard deviation was calculated, and the centered values were divided by this standard deviation.

**Figure 1:**
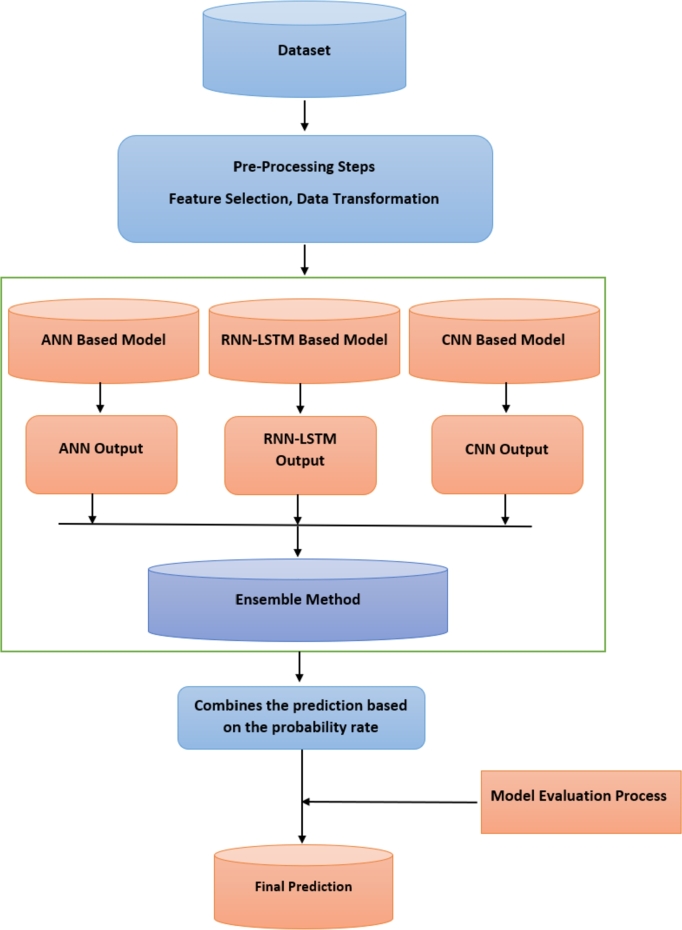
The diagram illustrates the flowchart for the ML-based method utilized in modeling path loss prediction.

Later on, the processed data was applied to each model, divided into training, testing and validating datasets based on a reliable hyperparameters tuning technique, namely Optuna [54]. This technique has the ability to find the optimum hyperparameters of the ML-based models (i.e., number of layers, number of neurons, type of activation functions, etc.) that lead to the highest possible prediction accuracy. More specifically, from the collected 865 samples, 80% of these samples were used for training and 20% for testing to validate the ensemble models' prediction accuracy, where All the results and figures are presented here as the output of the testing data. The ML-based path loss prediction strategy's flow chart is illustrated in Fig. 1.

### 2.3 Evaluation metrics calculation

Model evaluation metrics are mathematical equations applied to evaluate the performance of the adopted deep learning models and the proposed ensemble method. The evaluation metrics considered in this study are the benchmark for any regression problem that requires comparison with other algorithms within the same class. The five evaluation metrics used in this work are R-squared ($R^{2}$), root mean squared error (RMSE), mean absolute percentage error (MAPE), mean square error (MSE), and Pearson's correlation (Corr). These evaluation measures are extensively utilized in the research domain and are appropriate for algorithm comparison. Mathematically, these performance metrics can be defined as follows [43]:

(1) $$R^{2}=1-\frac{\sum_{j=1}^{M}{\left(S_{j}-\overset{ˆ}{S_{j}}\right)}^{2}}{\sum_{j}^{M}{\left(S_{j}-\overset{¯}{S_{j}}\right)}^{2}}.$$

(2) $$RMSE=\sqrt{\frac{1}{M}\sum_{j=1}^{M}{\left(S_{j}-\overset{ˆ}{S_{j}}\right)}^{2}}.$$

(3) $$MAPE=\frac{1}{M}\sum_{j=1}^{M}\left|\frac{S_{j}-\overset{ˆ}{S_{j}}}{S_{j}}\right|.$$

(4) $$MSE=\frac{1}{M}\sum_{j=1}^{M}{\left(S_{j}-\overset{ˆ}{S_{j}}\right)}^{2}.$$

(5) $$Corr=\frac{\sum_{j=1}^{M}(S_{j}-\overset{¯}{S})(\overset{¯}{S_{j}}-\overset{¯}{\overset{ˆ}{S}})}{\sqrt{\sum_{j=1}^{M}(S_{j}-\overset{¯}{S})(\overset{¯}{S_{j}}-\overset{¯}{\overset{ˆ}{S}})}}.$$

Where *M* is the number of samples used to determine the metrics values, *S* is the actual measured value of the path loss, and $\overset{ˆ}{S}$ is the predicted fitness value of the path loss. In contrast, $\overset{¯}{S}$ and $\overset{¯}{\overset{ˆ}{S}}$ are the mean values of the actual measured and the predicted values.

## 3 ML methods for path loss prediction

If incorporating machine learning methods in telecommunication or any other operation, the question that arises is *which machine learning methods should I use?* due to the increased number of current machine learning architectures and the improvements made therein. Each alternative method has its advantages and drawbacks. The motivating idea of this study is to combine the advantages of three models based on the ANN, RNN-LSTM, and CNN architectures in one method instead of selecting only one of these. This method was hypothesized to achieve a more optimal model with better accuracy than the individual models. The proposed ensemble method considers the advantages of these three ML-based models by training them with the same dataset to predict the path loss. In the ensemble step, we used a novel probability aggregation method based on applying a coefficient to the output of each model. The probability coefficients of the models were determined during the validation phase. Fig. 1 shows a flowchart of the ensemble method, which starts with pre-processing steps to prepare the dataset and select the best features as an input. Training the selected neural network models is performed. After that, the probability coefficients of these models are determined by searching for the best rate for each model. Finally, the ensemble method combines the sum of selected models multiplied by their appropriate probability rate.

The three models (ANN, CNN, and RNN-LSTM) have been selected for the ensemble method because they have shown high performance in previous work and are known to be successful and efficient methods for solving various problems. In our previous work, various machine-learning models have been adopted using the same dataset, and the results showed that the RNN-LSTM and ANN models have excellent performance [43]. The CNN model was chosen because it has been widely used in various applications and has shown to be a successful and efficient method for solving multiple problems and their ability to automatically learn and generalize to similar tasks without needing further learning. These motivated our study to select the models mentioned above to propose the ensemble method. Overall, the justification suggests that the three models were selected for the ensemble method because they have demonstrated high performance and are known to be effective and efficient methods for solving various problems [46].

### 3.1 ANN model

Artificial neural networks (ANNs) have been designed based on biological concepts. In particular, neurons have inspired the development of neural networks. The ANNs have various types of networks based on the structure of the network and the learning progress. They have been applied widely to solve challenges in various applications. The application of this study is supervised learning, where the inputs and the output are known for the training process. This study focuses on applying the ANN model with feed-forward learning techniques to predict path loss in [43]. The ANN model contains four hidden feed-forward neural networks. More discussion and various aspects of the ANN model used in this study are presented by us in [43]. In that publication, all structures of the adopted ANN model are described, including hyperparameter tuning, number of layers, number of neurons, learning rate, epochs number and sensitivity analysis.

### 3.2 RNN-LSTM model

LSTM networks are an upgraded version of the recurrent neural network (RNN) that applies alternative units to the standard units [55]. LSTM has special units called memory cells, which can keep information stored for long periods. The cells are applied to control and select the extracted features that enters the memory cells. Each cell includes three different gates: input, output, and forgets. The input gate is used to determine which information extracted from the last simple should be kept in the memory cell. In contrast, the output gate regulates the amount of data passed to the next layer, and forget gates control the tearing rate of memory stored. This architecture allows for learning longer-term dependencies.

Recurrent neural networks (RNNs) are a type of neural network that can process sequential data, such as time series data. Long short-term memory (LSTM) is a type of RNN designed to remember long-term dependencies in sequential data. The behavior of RNN-LSTM, when used with non-time series data, will still function as a regular feedforward neural network. This means it will take input data, process it through the network, and produce an output. However, it will not be able to use the sequential nature of the data, and it will not be able to remember long-term dependencies. For instance, if an RNN-LSTM is trained on a classification task using non-time series data, it can make predictions based on the input data. However, it will not be able to use information from previous inputs to improve its predictions. On the other hand, if the same RNN-LSTM is trained on a time series classification task, it can use information from previous time steps to make more accurate predictions. However, most of their best-known applications have shown excellent performance in non-sequential data situations where every training or testing case is independent [1], [2], [3]. This research adopted the RNN-LSTM [43] model using non-sequential data to extract feature representations that encode some aspects of the path loss.

This study used a similar RNN-LSTM architecture as given in [43]. This RNN-LSTM model includes the hyperparameters of two hidden layers of LSTM and feed-forward neural networks. The first hidden layer has 128 neurons while the last contains 32 neurons, each hidden layer is followed by a rectified linear unit (ReLU). The model is trained using a learning rate of 0.0001. The input layer of the neural network accepts four features extracted from the measured data, while the output layer consists of a single neuron with a linear activation function that yields an accurate value, which represents the path loss, utilizing the cost function to optimize the mean square error. More details on the RNN-LSTM used here for predicting path loss are to be found in [43].

### 3.3 CNN model

Convolution neural networks (CNNs) are well-known methods used to solve supervised and unsupervised problems [56], [57]. They have been shown to significantly improve solving challenges in various sectors, including medical imaging, telecommunication, etc. The CNN is an artificial neural network utilized to identify, extract, and examine intricate features. It comprises a set of layers with neurons that receive and process inputs consisting of high-dimensional vectors. To attain the best performance for the model, CNN employs several hyperparameters, which can be classified into two categories: those that determine the network structure and those that determine the network's training.

Hyperparameters are essential in determining a neural network's structure and training. Regarding the network structure, hyperparameters including kernel size, kernel type, stride, padding, hidden layer, number of neurons, and activation functions play significant roles. Kernel size determines the filter's dimensions, while kernel type reveals the filter's values (e.g., edge detection or sharpening). Stride dictates the frequency at which the filter moves over the input image, and padding adds layers of 0's to the image edges to ensure complete filtering. The number of neurons in each hidden layer and the activation functions allow the model to learn and predict nonlinear boundaries accurately.

As for the network's training, hyperparameters including learning rate, momentum, number of epochs, and batch size are crucial. The learning rate regulates the weight update after each batch, while momentum regulates the influence of previous weight updates on the current ones. The number of epochs signifies the number of iterations of the entire training dataset presented to the network, while the batch size refers to the number of input patterns shown to the network before weight adjustments occur.

The CNN has become a popular tool for analysis and prediction in machine learning. CNN is influential in several tasks, including image recognition, sentiment analysis and prediction problem [43], [56], [57]. Typically, a CNN receives input in a two-dimensional format, presented as a matrix or field. However, modifying the CNN's input format to be one-dimensional is possible, enabling the network to generate an internal representation of a one-dimensional sequence. It also can deal with three 3-dimensional input data. Generally, CNNs refer to a 2-dimensional CNN used for image analysis [58]. However, various research has been done using other input data types and has shown a successful performance. This work focuses on training a 1-dimensional CNN (1D CNN) using carefully selected input data. Since the input data is 1-dimensional, the approach utilizes 1D CNN to analyze and evaluate the learned weights during training and testing [59].

A 1D CNN is a type of CNNs that is designed to process data in a 1-dimensional structure, such as table data, which is similar to regular CNNs, but they use 1-dimensional filters instead of 2-dimensional filters, where they slide the filters along the length of the input sequence instead of over the spatial dimensions of an image. Since the proposed model uses a 1D CNN with a table database, the data was transformed into a 1-dimensional structure that the 1D CNN can process. After that, the 1D CNN can be applied to the data similarly to how it would be applied to a time series or other sequential data. The 1D CNN would use 1-dimensional filters to extract features from the input data, and these features would be used as input to the next layer of the network. The output of the 1-D CNN would depend on the specific task that the model is being trained for, such as regression or classification. Overall, a 1D CNN can be used with a table database by converting the data into a 1-dimensional structure and applying the 1D CNN to the transformed data. This can allow the 1D CNN to learn complex relationships between the elements in the sequence and make predictions based on the input data [59], [60].

Several reasons can justify why a 1D CNN is an excellent choice for processing our task and can be part of the ensemble method [59], [60]:

1 1D CNNs are specifically designed to handle sequential data, making them well-suited for processing data arranged in a 1-dimensional structure like a table.
2 1D CNNs can learn complex relationships between the elements in a sequence, which can help model the relationships between different columns in a table.
3 1D CNNs can be trained to perform various tasks, such as regression, making them a flexible choice for many data analysis tasks.
4 1D CNNs can be used in conjunction with other neural network architectures, such as recurrent neural networks (RNNs) or feedforward neural networks, to build hybrid models that can take advantage of the strengths of multiple types of neural networks.
5 A 1D CNN is relatively simple to implement and can be trained efficiently using standard machine learning techniques, making it a practical choice for many data analysis tasks.

Optimization of CNN hyperparameters is challenging due to the large number of hyperparameters in a standard CNN's architecture [61], and finding the optimized CNN model can improve CNN performance. This work adopted an optimized CNN model using the Optuna technique [54], which is shown to effectively select the optimal hyperparameters to achieve path loss prediction. The hyperparameters of our CNN model include two hidden layers. The first layer is a one-dimensional convolution layer with 128 filters and a kernel size of 2; the second is a feed-forward neural network layer containing 64 neurons. The ReLU activation function follows each hidden layer. The network input comprises four features extracted from the preprocessed data, which are the distance, frequency, antenna height, and angle of arrival (AoA). The output layer of the network consists of a single neuron, which utilizes a linear activation function as the transfer function to produce the predicted value. The Optuna technique determines the ideal values for hyperparameters that best fit the measured data. This approach ensures that the optimal values are obtained for precise model fitting, such as learning rate =0.001, batch size =32 and number of epochs =100.

### 3.4 The ensemble-method-based model

The proposed ensemble method has combined the benefits of the adopted ANN, RNN-LSTM, and CNN models into one model to predict the Path Loss for future wireless communication systems. The processed data is divided into training and testing datasets. After that, the Optuna technique selects the best hyperparameters for the adopted models [54]. This is done by training all models in several parameters and choosing the best hyperparameters that fit the measured data and minimize the prediction error as shown in Table 1. The best hyperparameters are used to train and test the models. The optimum results were obtained using a training size of 80%, while the test data size was 20%.

**Table 1:** Hyperparameters Tuning Configuration.

Models	Hyperparameters setting	Comments
ANN	# Hidden layers =4 # Neurons in 1st layer =96, 2nd layer =96, 3rd layer =32, and 4th layer =32. Activation functions are ReLU and Linear Learning rate =0.01 Optimizer = Optimizers.Adam Epochs number =50 Loss function is mean square error	These hyperparameters have been obtained using the Optuna technique, which has the ability to find the optimum hyperparameters of the ML-based models (i.e., number of layers, number of neurons, type of activation functions, etc.) that lead to the highest possible prediction accuracy.
RNN-LSTM	# Lstm layers =3 layers # Neurons in 1st layer =128, 2nd layer =64, and 3rd layer =32. Activation function are Tanh and Linear Learning rate =0.001 Optimizer = Optimizers.Adam Epochs Numbers =50 Loss function is mean square error	
CNN	One Conv1D layer # filters =128 filters, kernel size =2, activation function = ReLU. One MaxPooling1D layer with pool size =2 One Flatten layer with 128 neurons One Dense layer with 64 neurons and ReLU activation function. Learning rate =0.01 # of epochs = 50	
**Ensemble method**	Probability rate for each model after running all the possibilities value to obtain the corresponding values to the best result *β*_1_,*β*_2_,*β*_3_	

In regression applications, combining the predictions of a solution set is named ensemble learning, and the aggregation technique used is called the ensemble method. The proposed ensemble method combines and analyzes the results of three models: ANN, RNN-LSTM, and CNN. An averaging technique can be the most straightforward answer to combining the outputs of various models. The average result is a simple and effective method preferred in solution communities with close success scores. However, the proposed ensemble method requires that a robust method must greater impact on the final prediction when combining various models. In this study, this is achieved by giving more weight to the best model.

The ensemble method has been designed more efficiently here to predict path loss. Predicting path loss is challenging due to the reasons mentioned above. It is possible to solve these complex challenges with the weighted sum technique. The proposed ensemble method has achieved the goal by combining the outputs of each model with an optimization technique where the optimum weighted parameters can be obtained.

The proposed ensemble method multiplies the probability weight of each model with its model accuracy to predict path loss. These calculations can show the ensemble's path loss prediction using Eq. (6), where the $P_{i}$ notation is the probability weight of the *i* model. Since the softmax activation function is present in the last output layer of the three models, the sum of all probabilities given to each model is equal to one. The ensemble method used the training data to calculate the probability of each model in the ensemble. This probability is then used to weigh the individual models' predictions and combine them into a single ensemble prediction. Path loss predictions made by the three models using the training data are multiplied with their corresponding weighted value to give the ensemble method output. These multiplication results are then summed to produce the final ensemble model as depicted in Eq. (6), which can be used in testing data to produce path loss prediction.

(6) $$\text{Ensemble}\;\text{Output}=\sum_{i}^{3}P_{i}\times M_{i},$$

where $M_{i}$ is the path loss of the prediction results of each model.

## 4 Results and discussions

This section presents and discusses the main research finding of the paper. The results are based on plotting the predicted neural network models with the actual datasets individually to observe the behavior of each model in terms of fitting the measurement data. Moreover, the prediction models are plotted together with real data to provide a comparative analysis between the models. The comparison is also studied using the performance metrics mentioned above in equations (1)–(5). Furthermore, the run time and the input features' contribution to the accuracy of the models are numerically provided in this section.

**Figure 2:**
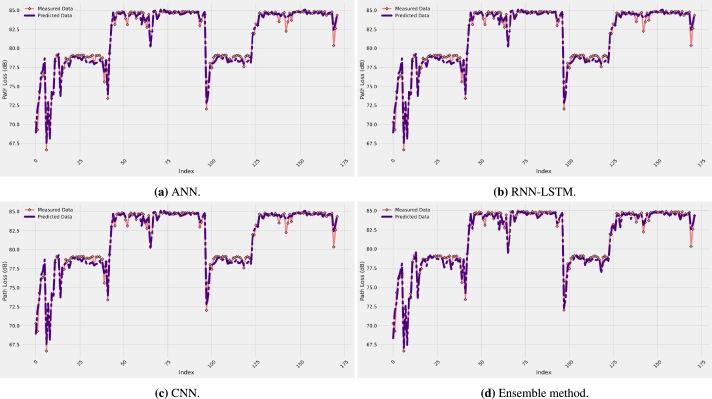
Prediction error curves of each model.

Measured data and the predicted path loss model results are given in Figs. 2a, 2b, 2c, 2d using the ANN, RNN-LSTM, CNN, and ensemble method, respectively, with four input features (distance, frequency, antenna height, and AoA). Generally, all the models accurately follow the measurement data. No overfitting and underfitting issues were found for all the models studied, and the training and testing of the models were performed several times to ensure the accuracy and stability of the results. As a justification of the results displayed in Fig. 2, the numerical results of the five performance metrics are presented in Table 2 for all the models with the four input features. For R-square values, a value of 0.9753 is found when the ensemble method model is used, which is the highest value and close to the ideal value of 1. The other models have achieved R-square values of 0.9352, 0.9160, and 0.9543 for the ANN, RNN-LSTM, and CNN models. This means that the R-square has improved by 4.3%, 6.5%, and 2.2% over the three models, respectively.

**Table 2:** Performance metrics' values of all the ML-based models selected.

Models	$R^{2}$			RMSE			MAPE			MSE			Corr		
	Min	Avg	Max	Min	Avg	Max	Min	Avg	Max	Min	Avg	Max	Min	Avg	Max
ANN	0.8625	0.9352	0.9665	0.0254	0.0355	0.0539	0.0221	0.0312	0.4535	0.0006	0.0013	0.0029	0.9523	0.9732	0.9870
RNN-LSTM	0.8863	0.9160	0.9470	0.0256	0.0418	0.0664	0.0234	0.0390	0.0530	0.0007	0.0019	0.0044	0.9315	0.9666	0.9869
CNN	0.9314	0.9543	0.9664	0.0267	0.0310	0.0478	0.0195	0.0263	0.0330	0.0007	0.0010	0.0023	0.9421	0.9781	0.9869
**Ensemble method**	0.9520	0.9753	0.9934	0.0102	0.0228	0.0324	0.0054	0.0204	0.0496	0.0001	0.0005	0.0010	0.9634	0.9884	0.9943

The best performance for the RMSE, MAPE, and MSE was achieved using the proposed ensemble since it provides the lowest values of these error metrics. However, all the models provide accurate path loss predictions since the maximum error value is less than 0.1 dB.

**Figure 3:**
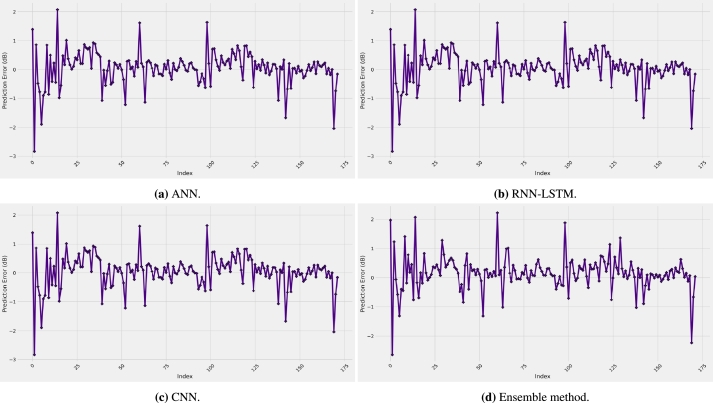
Prediction error curves of each model.

**Figure 4:**
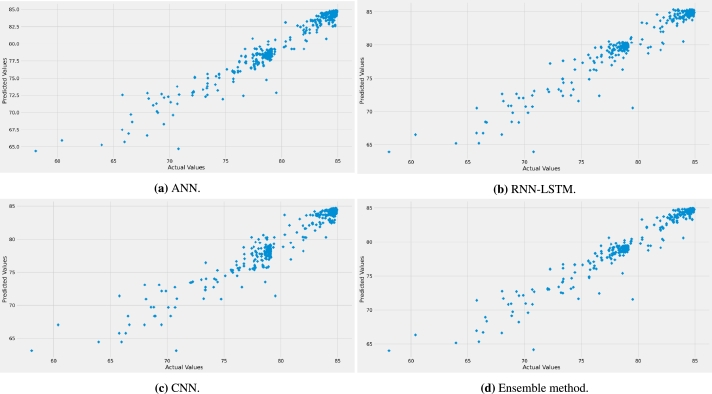
Predicted and measured path loss values for each model.

**Figure 5:**
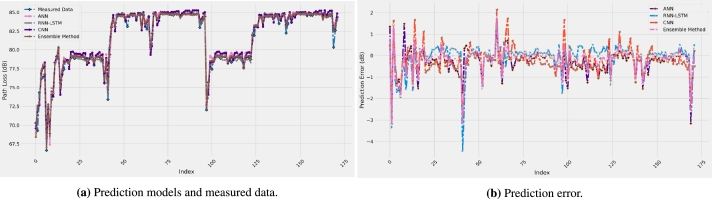
Path loss prediction models and prediction error for all the studied models together.

For the correlation coefficient, the best value is found to be 0.9884, achieved by the ensemble method model, whereas the worst value is 0.9666 when the RNN-LSTM model is used. Therefore, as a summary of the results presented in Fig. 2 and Table 2, the best prediction accuracy is achieved by the proposed ensemble method model. As a justification of the results, Figs. 3a, 3b, 3c, and 3d shows the behavior of the prediction error of all the four models selected. It is clear from the figure that the models exhibit relatively low values of prediction error since the distribution is mainly between -3 and 3 dB with the proposed ensemble method performing best. This is easily observable from Figs. 4a, 4b, 4c, and 4d for the four models selected, which depicts the predicted path loss values as a function of the real measured path loss values. Generally, all models provide a straight-line shape, meaning that the prediction path loss values are extremely close to the actual path loss values. The predicted models with the measurement data are represented together in Fig. 5a while the models prediction error are presented in Fig. 5b. These results are much better than using standard and improved empirical path loss models summarized in [6], [62], [63].

To evaluate the contribution of the antenna height as an input feature to the overall prediction accuracy of the neural network models, we removed it and kept the other three features (i.e., distance, frequency, and antenna height). As was expected, the prediction accuracy of all models is decreased. Specifically, the R-square value decreased by 4.5% for the ensemble method model, whereas the reduction is 6.7%, 3.2%, and 2.4% for the ANN, RNN-LSTM, and the CNN models. This means that the contribution of the antenna height to the overall model accuracy is not significant for enclosed indoor corridor environments where the richness of propagation mechanisms exists. Table 3 summarizes the performance metrics values after removing the antenna height from the input features of the models. From the table, the metric values do not have significant changes, which indicates the superiority of the distance, frequency, and the AoA as the main contributors to overall model accuracy.

**Table 3:** Performance metrics after removing the antenna height from the input features of the models.

Models	$R^{2}$			RMSE			MAPE			MSE			Corr		
	Min	Avg	Max	Min	Avg	Max	Min	Avg	Max	Min	Avg	Max	Min	Avg	Max
ANN	0.8824	0.9093	0.9281	0.2555	1.7297	2.0492	0.0225	0.0801	0.6611	0.0653	2.9918	4.1992	0.9162	0.9382	0.9625
RNN-LSTM	0.8423	0.8509	0.8715	0.3925	1.4149	2.0356	0.0191	0.0893	2.3132	0.1541	2.0021	4.1437	0.8746	0.9062	0.9344
CNN	0.9123	0.9323	0.9526	0.0256	0.0347	0.3509	0.0190	0.0342	0.3132	0.0006	0.0012	0.1231	0.9446	0.9693	0.9787
**Ensemble method**	0.9588	0.9685	0.9823	0.0153	0.0243	0.0723	0.0156	0.0243	0.0975	0.0002	0.0001	0.0052	0.9588	0.9787	0.9850

We removed the antenna height and AoA from the models' input features. The performance metrics' results are presented in Table 4. Here, a notable change in the values reveals that the AoA contributes considerably to the models' prediction accuracy. Generally, the best performance is still observed when the ensemble model is used. However, as it depends on all the other three neural network models, the ensemble method model has a longer run time of 276.7636 seconds, almost 4.7 times the time required to run the ANN model. This ANN model requires a minimum time of 59.1485 seconds, among the others. Table 5 summarizes the run time required for the training process of each model.

**Table 4:** Performance metrics after removing the antenna height and the AoA from the input features of the models.

Models	$R^{2}$			RMSE			MAPE			MSE			Corr		
	Min	Avg	Max	Min	Avg	Max	Min	Avg	Max	Min	Avg	Max	Min	Avg	Max
ANN	0.7932	0.8482	0.8848	0.9742	2.0028	2.9048	0.0538	0.1049	0.8860	0.9491	4.0114	8.4378	0.8741	0.9224	0.9395
RNN-LSTM	0.8032	0.8240	0.8599	1.0684	2.0226	3.0206	0.0427	0.0998	0.7737	1.1414	4.0910	9.1240	0.8942	0.9278	0.9430
CNN	0.8832	0.9099	0.9207	0.0684	0.1019	0.1206	0.0735	0.6763	0.8556	0.0046	0.0107	0.0454	0.8058	0.8356	0.8942
**Ensemble method**	0.8944	0.9249	0.9523	0.0198	0.0993	0.1958	0.0480	0.0684	0.0992	0.0004	0.0098	0.0383	0.9023	0.9401	0.9631

**Table 5:** Runtime comparison of the adopted models.

Models	RunTime [seconds]
ANN	59.1485
RNN-LSTM	124.9184
CNN	92.6543
**Ensemble method**	**276.7636**

The complexity and run time of the model are increased compared to the other models. However, as a tradeoff between the model's complexity and accuracy, the ensemble method model provides the highest accuracy and stability among all the other three models and the other well-known ML-based models published in [43]. The proposed ensemble method model can be a trustful model to predict path loss for high-frequency bands in complex environments where the propagation signal suffers from several effects in the wireless channel.

The proposed ensemble algorithm combines ANNs, RNN-LSTMs, and CNNs, then the training time for the ensemble algorithm is likely the sum of the training times for each individual model, as indicated in Table 5. This means that the training time for the ensemble will be longer than the training time for any single model, which could result in a disadvantage in terms of high convergence speed. The cause of this disadvantage is the increased computational complexity of the ensemble algorithm resulting from combining multiple models. This increased complexity can slow the training process and reduce the algorithm's convergence speed. However, regarding implications for theory and practice, this disadvantage may limit the applicability of the ensemble algorithm in scenarios where training time is a critical factor. For example, if the training time for the ensemble algorithm is too long, it may not be practical to use it for real-time applications or large-scale problems. However, the ensemble algorithm may still be helpful for other types of problems where training time could be more critical or where the benefits of the ensemble approach outweigh the increased training time.

## 5 Conclusion

An ensemble method is proposed based on combining three deep learning techniques. The ensemble approach results have shown that the architecture can identify and predict a high number of the measured path loss data and minimize the mean square error. This conclusion was based on 20% testing performed on the measured dataset. It achieved a high accuracy percentage of the correlation and R-square metrics while reducing the mean square error, absolute mean square error and the root square error to a significant error. These results indicate that the proposed ensemble method can provide an accurate path loss prediction model, which can play a significant role in modeling wireless communication channel for 5G systems and beyond.

The ensemble method also integrates the advantages of combined models to overcome the shortcomings of each technique when used individually. This has been done to resolve issues related to one another when used for path loss prediction challenges. It also applies the Optuna technique or hyperparameters-tuning, which can look for the best hyperparameters values and the optimum probability weight for each model. Also, this architecture focuses on improving the limitation of deep neural networks in path loss prediction. The extraction of the skin path loss features correctly differentiates them from the image backgrounds. Thus, it is the best performing method considering these aforementioned results; the ensemble method outperforms most existing methods. The best performance of the proposed method is primarily because of the contribution of the combination of the three neural networks models (i.e., ANN, RNN-LSTM, and CNN).

Future directions after this research are apparent. Firstly, the proposed method presented in this work can be evaluated using different measured datasets to verify whether they are limited to data or can be generalized. To achieve that, measurement campaigns in higher frequency bands for different indoor and outdoor environments will be conducted to collect the required data for training and testing the ML-based models. Secondly, the proposed ensemble method can combine more machine learning methods in one model integration to detect the path loss with a high accuracy rate. Thus, it is worth investigating the integration of deep learning techniques with different models based on ensemble theory. These studies are of importance to achieve the best path loss models that can be reliable for wireless systems' planning and link budget calculations.

## Funding statement

This work is based on the research supported in part by the 10.13039/501100001321National Research Foundation of South Africa (Grant Numbers 131604).

## CRediT authorship contribution statement

**Mohamed K. Elmezughi:** Conceptualization; Data collection; Performed the experiments; Analyzed and interpreted the data; Coding; Wrote the paper. **Omran Salih:** Conceptualization; Performed the experiments; Analyzed and interpreted the data; Coding; Wrote the paper. **Thomas J. Afullo:** Conceived and designed the experiments, draft manuscript preparation. **Kevin J. Duffy:** Analyzed and interpreted the data; draft manuscript preparation. All authors reviewed the results and approved the final version of the manuscript.

## Declaration of Competing Interest

The authors declare that they have no known competing financial interests or personal relationships that could have appeared to influence the work reported in this paper.

## Data Availability

Data will be made available on request.
